# Architects of folding, editors of immunity: the strategic use of *N*-glycans in vaccine design

**DOI:** 10.3389/fimmu.2026.1766701

**Published:** 2026-02-18

**Authors:** Sofia Orioli, Tommy Idrovo-Hidalgo, Maria de los Angeles Martinez Saucedo, Cecilia D’Alessio

**Affiliations:** 1 Universidad de Buenos Aires, Facultad de Ciencias Exactas y Naturales, Departamento de Fisiología y Biología Molecular y Celular, Instituto de Biociencias, Biotecnología y Biología Traslacional (iB3), Buenos Aires, Argentina; 2 Consejo Nacional de Investigaciones Científicas y Técnicas (CONICET), Buenos Aires, Argentina

**Keywords:** antigen, glycoengineering, immunogenicity, *N*-glycosylation, protein folding, vaccine design

## 1 Introduction

Public health strategies rely heavily on vaccination to reduce the burden of infectious diseases (1–3). From 1974 to 2024, vaccination is estimated to have prevented 154 million deaths and contributed to 40% of the decline in global infant mortality (4), eradicating once-devastating pathogens (5, 6). The sudden emergence of highly transmissible pathogens can disrupt daily life, increase mortality, and severely affect economic growth, especially in low- and middle income countries (LMICs) (7–9). Several types of vaccines have been developed, with traditional formulations based on attenuated or inactivated pathogens (10–12). Subunit vaccines-based on pathogen proteins- offer a safer and versatile alternative when compared to conventional inactivated or attenuated vaccines, minimizing infection risks while eliciting protective immune responses (13).

Achieving robust and broad immunity depends not only on which pathogen regions are selected as antigens but also on how these regions are presented to the immune system. Immunodominant regions are often the most variable in sequence (14–16), or structurally hidden by post-translational modifications that help pathogens evade recognition (17). Among these modifications, protein *N*-glycosylation plays a central—and often paradoxical—role in antigen biology and recombinant vaccine antigen design. Enveloped viruses such as HIV-1 or influenza A bear *N*-linked glycans in their membrane proteins and use the host’s glycosylation to shield important epitopes (18, 19). Notably, *N*-glycosylation contributes to both viral immune evasion and glycoprotein folding and ER quality control, albeit through distinct glycan structures (20). In patients with congenital disorders of glycosylation such as MOGS-CDG-a disorder caused by mutations in glucosidase I, the first enzyme involved in glycan remodeling after transfer to proteins – the replication of many enveloped viruses is reduced (21, 22). This dual role of *N*-glycosylation poses a challenge for vaccine development: producing cost-effective antigens while ensuring proper folding and immunogenicity. These underscore the need to conceptually separate the folding and immunogenic functions of *N*-glycans. Viewing glycans as tunable design elements opens new opportunities for rational, cost-effective, and globally accessible vaccine development. In this article we dissect the roles of *N*-glycosylation in rational antigenic design.

## 2 Roles of *N*-glycosylation in antigen folding and immunogenicity

### 2.1 *N*-glycans and glycoprotein folding

*N*-glycosylation is one of the most frequent post-translational modifications of the secretory pathway: a preassembled oligosaccharide–conserved in mammals, yeast and plants- is added to the consensus sequence N-X-S/T (X cannot be P) of proteins that are entering the endoplasmic reticulum (ER). *N*-linked glycans participate in protein folding in the ER, 1) by increasing solubility and preventing aggregation of folding intermediates (23, 24), and 2) by allowing the protein interaction with the ER quality control of glycoprotein folding (ERQC) (25, 26). This process ensures that only properly folded glycoproteins proceed to secretory pathway, while misfolded proteins are retrotranslocated to the cytosol and degraded (27).

Several studies have demonstrated the critical role of *N*-glycosylation in the correct folding of recombinant proteins. For example, expression of the receptor-binding domain (RBD) of the SARS-CoV-2 spike protein in *Nicotiana benthamiana* showed that *N*-glycosylation is essential for proper folding, as mutants generated by site-directed mutagenesis of glycosylation sites could not be produced as soluble proteins (28). Yields of Receptor Binding Domain (RBD) from SARS-CoV-1 expressed in *Pichia pastoris* decreased with the reduction of *N*-glycosylation sites (29). Deglycosylated *Aspergillus niger* α-L-rhamnosidase (r-Rha1), produced in *P. pastoris* either by *in vivo* inhibition of *N*-glycosylation or by *in vitro* enzymatic deglycosylation, revealed that *in vivo* inhibition led to greater structural destabilization and a more pronounced loss of enzymatic activity (30). These examples highlight the importance of glycosylation during folding, although individual *N-*glycosylation sites contribute differently to folding, trafficking or protein function (31, 32).

### 2.2 *N*-glycans and immunogenicity of antigens

Once folded glycoproteins leave the ER, their *N*-glycans undergo species-specific remodeling along the secretory pathway, generating the diversity found in mature proteins: high-mannose, complex, or hybrid forms (33). *N*-glycans are crucial in host-pathogen and cell-cell interactions and both pathogenicity and infection resistance can depend on glycosylation of secreted or surface proteins (34–38).

Immune-evasion mechanisms, such as masking of conserved epitopes, frequently rely also on glycosylation (39–42) and secreted effector glycoproteins, which may need to be glycosylated to be active and contribute to virulence (43, 44). For example, the *Haemophilus influenzae* surface glycoprotein HMW1 mediates host-cell adhesion via a membrane-anchored glycan but upon deglycosylation, adhesion is lost (36, 45). HIV, SARS-CoV-2, and Influenza are enveloped viruses whose surface glycoproteins mediate receptor binding and entry, making them major vaccine targets (46, 47). However, evolutionary pressure drives immune-evasion through continual mutation of these immunodominant proteins (48). In influenza, immune response focuses on hemagglutinin (HA), but antigenic drift accumulates mutations, undermining vaccine effectiveness (49, 50). HIV-1 vaccine development faces similar obstacles: the virus has high mutation rates, while heavily glycosylated Env protein shields key epitopes (51–53). For SARS-CoV-2, the immune response mainly targets the Spike glycoprotein, which contains 22 glycosylation sites per monomer (54, 55).

The high structural variability of carbohydrates confers proteins exceptional diversity (56). Their role in immune evasion complicates antigen selection for vaccines (57). Emerging glycoengineering approaches may help overcome these challenges and improve vaccine design.

## 3 Glycoengineering in vaccine designs

**Figure 1:**
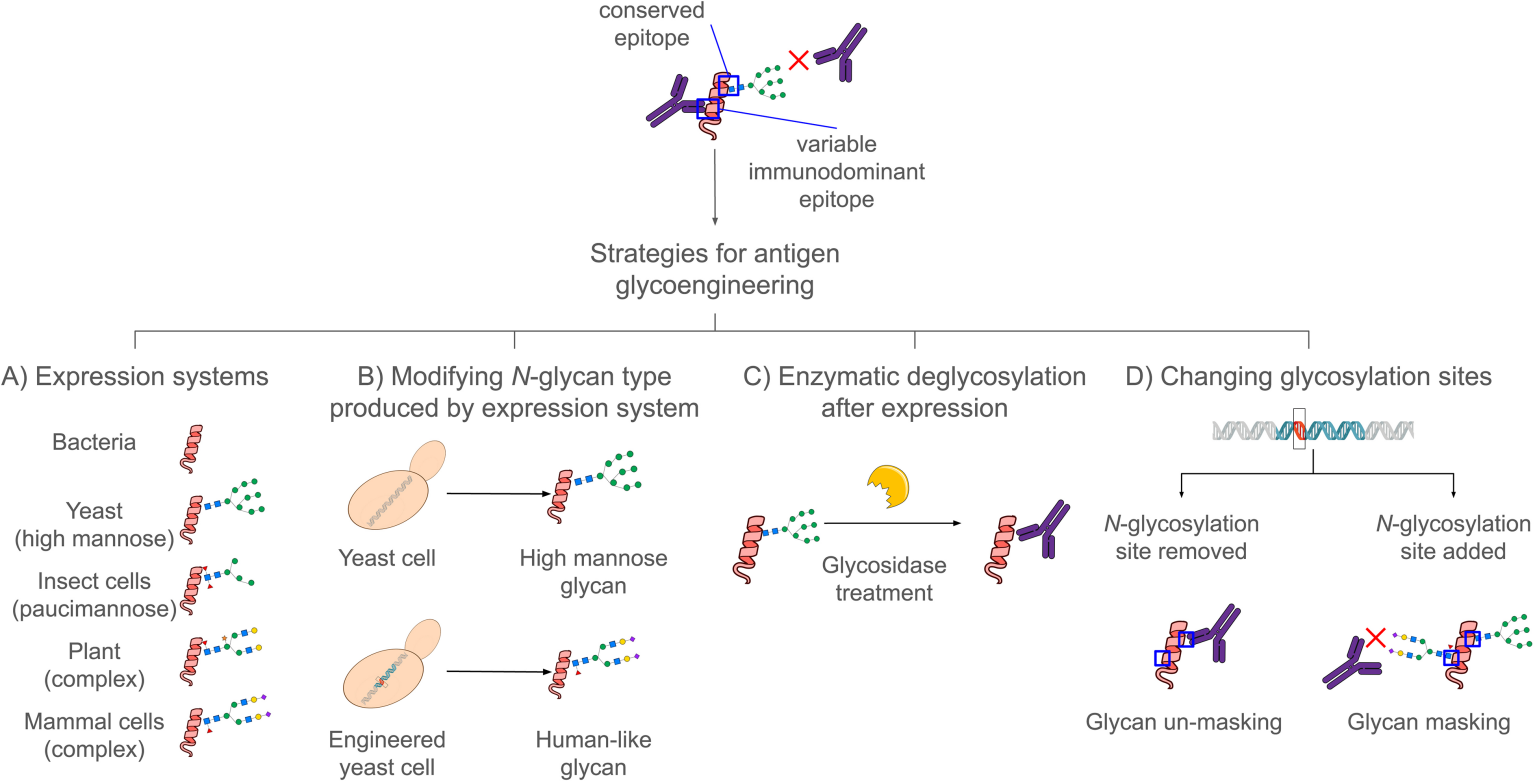
Strategies in antigen glycoengineering. The monosaccharides follow the Symbol Nomenclature for Graphical Representations of Glycans (58). The enzyme-yellow icon was adapted from Servier Medical Art (https://smart.servier.com/), licensed under CC-BY 3.0 Unported. All other icons were obtained from BioArt and BioIcons under their respective open licenses.

Glycoengineering strategies for improving vaccine antigens involve optimizing the carbohydrate structures attached to proteins to improve immunogenicity, safety, or production yield. As *N*-glycans influence folding, trafficking and immune recognition, their modification provides a unique opportunity to explore their roles in diverse contexts, expanding vaccine design possibilities. Below, we outline and critically assess the main glycoengineering strategies used to enhance antigen’s performance (**Figure 1**).

### 3.1 Strategy A — choosing the expression system

The glycosylation pattern of a recombinant protein is determined by the host system used for its production (59, 60). Consequently, selecting an expression platform is inherently a form of glycoengineering, as the host dictates the glycan repertoire displayed on the antigen (59, 60). *E. coli* adds no *N*-glycans; mammalian cells typically produce complex-type glycans; insect cells generate high-mannose and paucimannose structures; plant cells yield biantennary GlcNAc-terminating *N*-glycans; and yeasts are characterized by extensive high-mannose *N*-glycans (61). For SARS-CoV-2 Spike protein, the complex-type glycans produced in CHO cells correlate with stronger neutralizing antibody responses in mice when compared to Spike produced in yeast or insect cells (62). Although mammalian cell culture remains the gold standard for producing biotherapeutics, alternative expression systems may offer complementary advantages depending on the antigen and manufacturing context, such as higher expression yields, access to distinct post-translational modifications, or increased process flexibility compared with the native host (63). However, each organism imposes a characteristic “glycan signature” that can alter protein function (56) or elicit an undesired immunogenic response often requiring additional engineering to mitigate these effects (64–66). Thus, understanding system-specific glycosylation is fundamental to subsequent engineering decisions, and the choice of expression host should also consider potential drawbacks, such as the need to remove lipopolysaccharide (LPS) endotoxins from recombinant proteins produced in *E. coli* for animal or human use (67).

### 3.2 Strategy B — modifying the N-glycosylation pathway of a cell type

Several strategies aim to homogenize or “humanize” *N-*glycans in eukaryotic systems such as yeast and plants. For instance, secreted IgG1-Fc with truncated *N-*glycans extendable into diverse structures was produced in a *P. pastoris* expressing a Golgi-localized endoglycosidase Endo T (68). This GlycoDelete strategy has also been applied in plants, whose glycoproteins normally contain heterogeneous forms with β-1,2-xylose and core α-1,3-fucose, sugars recognized by human IgG1 in many non-allergic blood donors. To eliminate these residues, a Golgi-targeted Endo T from *Hypocrea jecorina* was expressed in seeds of an *Arabidopsis* GnTI mutant. The resulting recombinant activation associated secretory protein 1 (ASP1) glycoprotein produced carried single GlcNAc residues, showing that GlycoDelete can be used for other recombinant proteins not requiring complex *N-*glycans while removing immunogenic plant sugars (69). A successful example of *N*-glycan engineering in bacteria is the introduction of the *Campylobacter jejuni N*-glycosylation pathway into *E. coli*, enabling the production of glycoproteins bearing a heptasaccharide on D/E-X-N-X-S/T sequons (70).

Another approach to glycoengineering in *P. pastoris* is GlycoSwitch technology. The SuperMan5 strain -engineered to prevent hyperglycosylation and to shift the glycan profile toward Man_5_GlcNAc_2_- enabled methanol-independent production of an IgG Fc with a more homogeneous and size-defined glycosylation pattern. Although these glycans are not complex and do not correspond to the typical Fc glycosylation of human IgG1, their reduced heterogeneity may offer advantages from a production and quality control perspective, positioning this platform as a potentially safe and cost-effective alternative to mammalian cell culture for antibody manufacturing (71). A glycoengineered *P. pastoris* strain capable of producing fully complex and terminally sialylated *N*-glycans has been successfully used for the expression of functional erythropoietin, a glycoprotein whose efficacy and receptor affinity critically depend on its glycosylation profile (72).

Nonetheless, switching *N*-glycan types in a host requires careful consideration: deleting or introducing entire pathways may compromise genetic stability (73) and enzymes introduced to manipulate the glycosylation pathway may be deletereous on the host organism (70). Further challenges include the time-consuming optimization needed to ensure enzyme activity in the host environment and the difficulty achieving fully uniform *N*-glycan profiles (68).

### 3.3 Strategy C — post-expression deglycosylation: folding first, glycan removal later

Another strategy to modify an antigen’s *N-*glycosylation profile is to remove or modify glycans enzymatically. This approach relies on the observation that many antigens require glycans for proper folding but do not require them for immune presentation. By allowing *N-*glycans to assist protein folding and then eliminating them after purification, previously shielded conserved epitopes can be exposed and size heterogeneity eliminated. Following this rationale, the SARS-CoV-2 RBD was produced in *P. pastoris* with its two native *N-*glycosylation sites intact during expression, followed by enzymatic deglycosylation with Endo H after purification (74). The resulting deglycosylated antigen maintained structural integrity while presenting to the immune system or patient’s serum a homogeneous surface with a single GlcNAc remaining, thus avoiding the heterogeneity of yeast high-mannose glycans. This strategy has also been applied in plants, where RBD and the malaria antigen Pfs48/45 were produced in *Nicotiana benthamiana* co-expressing bacterial Endo H, enabling *in vivo* deglycosylation (75, 76). A similar approach was used for the *Bacillus anthracis* protective antigen (PA), a promising candidate for a cost-effective and immunogenic anthrax vaccine (77).

Although the post-expression deglycosylation strategy is useful to produce recombinant antigens in a low-cost scalable host system without non-mammalian glycosylation, several issues need to be evaluated. It is known that some deglycosylation enzymes, such as PNGase F, can remove complex glycans but also deamidates asparagine residues, potentially altering protein structure (77). In addition, certain antigens, as the APA complex of *Mycobacterium tuberculosis* experience up to a ten-fold activity loss in eliciting delayed-type hypersensitivity reactions in guinea pigs immunized with BCG upon deglycosylation, indicating that their mannose residues may be necessary to maintain antigenic properties (78). Deglycosylated proteins may be more aggregation-prone, less thermally stable, and more susceptible to proteolysis (79), along with potential structural alterations (30). In addition, the manufacturing costs associated with the inclusion of a deglycosylation step should be taken into account.

### 3.4 Strategy D — changing glycosylation sites for masking and unmasking epitopes

Another approach involves adding or removing *N-*glycosylation sequons to reshape which regions of an antigen are targeted by the immune system. Glycan masking works by sterically shielding undesired, variable, or immunodominant epitopes, redirecting antibodies toward conserved or functionally important regions. Conversely, glycan unmasking reveals epitopes normally hidden during infection, enabling recognition of vulnerable structural features pathogens often conceal.

These strategies have been applied to several viral antigens. In HIV, *N-*glycans introduced in Env variants (eOD-GT8) focused immunity on the CD4 binding site (80). For Influenza H5N1, masking hypervariable HA regions broadened cross-clade neutralization (81, 82), with further gains when multiple masked immunogens were combined (83). Pairing head masking with stem-epitope unmasking also produced cross-clade protection in mice (81–84). Similar glycan-based immune-focusing approaches have been explored for Ebola glycoprotein (85) and SARS-CoV-2 RBD (46), highlighting the versatility of rational glycan design.

Epitope redirection via glycan masking and unmasking is not without risks. Adding *N*-glycans can induce conformational changes that destabilize the antigen or alter the native epitope architecture (86, 87). Removing glycans can reduce thermal stability, increase aggregation, or proteolytic susceptibility.

## 4 Discussion

Current glycoengineering strategies show that the key question in antigen design is not whether glycosylation is present, but *when* during biosynthesis it is required and *for what function*. Glycans often serve as essential biosynthetic elements that support folding and quality control, yet they may hinder immune recognition once the protein reaches its mature state. Our opinion is that glycosylation should be viewed as a tunable design parameter, necessary during glycoprotein biosynthesis but amenable to rational remodeling or removal in the final recombinant immunogen. This perspective shifts glycoengineering from merely replicating native viral glycosylation toward strategically redesigning it.

Within this framework, the choice of the expression systems and downstream processing becomes a deliberate design decision. The predictable glycosylation patterns of host cells enable the use of glycans as temporary folding aids while allowing controlled optimization of epitope exposure. Overall, effective vaccine design should rely on distinguishing when *N*-glycans are biologically essential and when they are immunologically dispensable.
